# "The era of single disease cowboys is out": evaluating the experiences of students, faculty, and collaborators in an interdisciplinary global health training program

**DOI:** 10.1186/s12992-018-0343-z

**Published:** 2018-03-01

**Authors:** Anna Kalbarczyk, Nina A. Martin, Emily Combs, Marie Ward, Peter J. Winch

**Affiliations:** 1 Johns Hopkins Bloomberg School of Public Health, 415 N Washington St, Baltimore, MD 21231 USA; 2 University of Washington, 2301 Fifth Ave, Suite 600, Seattle, WA 98121 USA; 3 Population Services International (PSI), 1120 19th St NW, Suite 600, Washington, DC 20036 USA; 4 Johns Hopkins Bloomberg School of Public Health, 615 N Wolfe St E5533, Baltimore, MD 21231 USA

**Keywords:** Interdisciplinary, Global health, Training, Mentorship, Education

## Abstract

**Background:**

Global Health is an inherently interdisciplinary field but overseas training in global health, particularly among health science institutions, has been an ‘individual’ or ‘individual discipline’ experience. Team-based training is an approach to global health education which is increasing in popularity; research on team-training demonstrates that teams are more productive than individuals. In 2015, the Johns Hopkins Center for Global Health (CGH) developed the Global Established Multidisciplinary Sites (GEMS) program, an interdisciplinary training program which was designed to establish a new norm in global health training by bringing interdisciplinary teams of faculty and students together to identify and solve complex global health challenges. This research aims to evaluate the program’s first year and contribute to the literature on interdisciplinary team training. We conducted 22 in-depth interviews with students, faculty, and local collaborators from 3 GEMS project sites. Findings were analyzed for themes through a framework approach.

**Results:**

The program exposed students, faculty, and collaborators to a wide range of disciplines in global health. Students’ desire to learn how other disciplines contribute to global health solutions was an important motivator for joining GEMS; many participants including faculty and collaborators valued exposure to multiple disciplines. Mentorship and communication were a challenge across all teams in part due to members having distinct “disciplinary languages”. Balancing disciplinary representation on teams and establishing work plans were also key challenges.

**Conclusions:**

Based on the data the CGH provides four recommendations for institutions developing global health interdisciplinary teams to optimize team functioning and address challenges in mentorship, language, and roles: 1) address interdisciplinary communication early, 2) develop work plans during group formation, 3) meet as a team prior to travel, and 4) establish regular check ins. This article provides first-hand reflections on interdisciplinary team experiences in a global context and provides a pathway for the development of innovative strategies in global health training.

## Background

Global health is an inherently interdisciplinary field, spanning medicine, nursing, public health, pharmacy, engineering, business, anthropology, political science, and other fields. In a recent survey of 41 postgraduate global health programs, Withers et al. found that many of the programs were housed overwhelmingly in schools of public health or medicine, and recommended that programs include disciplines such as the social sciences to make the global health education more comprehensive [1]. Many institutions emphasize the importance of multiple discipline learning and have built this into core centers and program missions. Columbia’s Next Generation Internships program is a multidisciplinary training initiative drawing students from across disciplines; students work integrally with global health experts for two to six months on ICAP-supported programs [2]. Harvard, Duke, and the University of Toronto, among many schools, both have organizing offices which encourage and support multidisciplinary partnerships though the mechanisms for student engagement are unclear [3–5]. While this is not a comprehensive list, it serves to highlight the increased prevalence and recognized need for multiple discipline global health programs.

However, overseas training in global health, particularly among health science institutions, has been an “individual” or “individual discipline” experience: Adams et al. discuss the variety of pathways available to individuals today to be engaged in global health, ranging from “extended structured internships for students or trainees to shorter-term teaching or specialty practice opportunities for faculty” [6]. Such models typically rely on one-on-one interactions between a more-experienced “mentor” and a less-experienced “mentee”, a form of what Dennen describes as “cognitive apprenticeship” [7]. In the context of global health education, this closely resembles practicums, internships, or dissertation fieldwork activities which are part of degree requirements. During these activities, a student would work with one or more faculty members in a designated area of mutual interest for a period of time to achieve specific goals. While such an arrangement allows for close contact and sharing of knowledge between mentors and mentees and can result in significant impacts across a range of academic, social, and emotional areas [8], it may also be, as Oberhelman et al. note in their review of postdoctoral training programs, “…at odds with current trends in higher public health education, which increasingly emphasize the value of interdisciplinary training, translational science, and solution-oriented, experiential learning—that is, interdisciplinary training that encourages researchers to evaluate public health problems and potential solutions from multiple perspectives” [9].

A further, increasingly popular approach to global health education is team-based training, which we define as individuals from different disciplines joining together to achieve a common goal. Research on team-training demonstrates that teams are more productive than individuals. There are documented benefits to interdisciplinary, team-based training. Teams tend to produce more highly cited work than individual authors and research with higher impact [10]. Another study found that trainees who participate in team-based training better learn “how to respect the value and values of others and to worry less about submerging their professional identity in the team process” [11].

Team-based training is not without challenges. The inclusion of multiple perspectives can result in increased decision-making time and additional resources [12]. Team dynamics, i.e. the amount of time each member speaks or sensitivity to other members’ social cues, and the amount of “psychological safety” created, i.e. freedom to express ideas without fear of rejection [13], influence the success of a team [14]. However, there is a limited understanding about what influences team effectiveness and how to build a team which produces quality outputs [15–17], particularly in the context of global health training.

The Johns Hopkins Center for Global Health (CGH) has supported individual global health training experiences for more than a decade. In 2015 the CGH developed the Global Health Established Multidisciplinary Sites (GEMS), an interdisciplinary training program which aimed to establish a new norm in global health training by bringing interdisciplinary teams of faculty and students together to identify and solve complex global health challenges. GEMS sites are expected to foster three core activities: build interdisciplinary teams of students and faculty; foster established relationships with international collaborators and field sites; nurture a program and funding model that emphasizes the “longer term”. To support this latter activity, the CGH provides funding to each site to develop institutional infrastructure to support training programs (i.e. coordination costs, salary support for local administrators, etc.) in addition to student grants. In its pilot year, 3 GEMS were created: one in Bangladesh, one in India, and one in South Africa. The program provided travel and living support to 14 trainees and incorporated the expertise of 11 faculty across the University, representing five of the nine Hopkins schools (Bloomberg School of Public Health, Carey Business School, Krieger School of Arts and Sciences, School of Medicine, and School of Nursing).

Hopkins faculty and their collaborators recruited team members through an application process facilitated by the CGH, through pre-existing relationships, and through word of mouth. Faculty were required to assemble a team of students representing no fewer than three of the nine JHU divisions (e.g. medicine, business, and public health). Each team worked together in Baltimore, Maryland prior to departure and traveled overseas at different times throughout the program year, depending on team and collaborator availability. Faculty and local collaborators were encouraged to determine when and how frequently each student would travel, though it was expected most would go as a cohort. Students were expected to be in-country for no less than 4 weeks, and these trips could happen at multiple times over the course of one calendar year.

Many terms have evolved to describe contributions from different disciplines: in a review of nursing education literature, Dyer describes Garner and Hoeman’s definition of “interdisciplinary” work as work that emphasizes the collaborative nature, beyond the common goals and objectives of “multidisciplinary” work [18]. The GEMS program has evolved from considering each team’s work as a shared multidisciplinary process to better capture what Stokols et al. describe as an “interdisciplinary” approach [16]. At each site, teams of students and faculty from separate disciplines (e.g. Engineering, Nursing, and Anthropology) work together and draw on their respective disciplines to reach an overall project goal.

This research aims to evaluate the first year of this global health team-based training program and contribute to the literature on interdisciplinary team training. Specifically, our evaluation sought to better understand how students, faculty, and collaborators perceive the nature of collaboration within interdisciplinary teams and how to optimize the team learning experience and functioning.

## Methods

We sampled 25 students, faculty, and local collaborators from three GEMS sites for in-depth interviews (IDI). Participants were sampled based on availability during visits to the field sites, seeking to interview as many members of each team as possible during the field visits. At a minimum, we aimed to interview one representative of each discipline participating in the team. Interviews were conducted by CGH staff at GEMS sites and at the Johns Hopkins University campus, depending on interviewee availability. Semi-structured interview guides for students covered personal history with the project, individual contributions, nature of interdisciplinary collaborations, and working with mentors/collaborators. Interview guides for faculty and collaborators covered personal history with the project, student team interactions, and mentorship experiences. All interviews were conducted in English, audio-recorded by digital recorders and transcribed by CGH staff. Findings were analyzed for themes using a framework analysis approach. Study team members read the transcripts and then discussed and developed a set of codes to be used in matrices. Matrices were organized to reflect both the interview guide and the emerging themes from the data. The organization of matrices was applied by all team members to a sample set of transcripts which reflected the range of interviewee types. The sample matrices were reviewed, refined, and finalized by the study team. The final matrix was then applied to all other transcripts from the study.

Prior to the interview student participants were also asked to complete a quantitative ranking exercise where they assigned 19 different programmatic aspects a difficulty level of 0–100 (0 being not at all difficult and 100 being very difficult). Program aspects included adapting to local styles of work and administrative procedures, and understanding concepts, theories, and methods from other disciplines or areas. The research team elected to only provide the quantitative ranking exercise to students because faculty mentors and collaborators were not all equally involved in the many of the training aspects.

## Results

We conducted 22 interviews between July, 2015 and January, 2016 with 14 students, 3 faculty, and 5 local collaborators from three GEMS sites who were available at the time of interview. Table 1 lists the distribution of students by discipline and site.

**Table 1:** Student Disciplinary Distribution Across Sites

	Anthropology	Business	Engineering	Informatics	Medicine	Public Health	Total
Bangladesh			4	1	1		6
India		2				1	3
South Africa	1	1	1			2	5

GEMS teams traveled internationally at different times and for different durations depending on team member availability. On average students spent between 4 and 12+ weeks at a site, depending on the site’s needs.

When asked why they chose the GEMS program, every student stated that s/he wanted to learn how other disciplines functioned and approached the global health challenge. Most students stated that the project they chose appealed to them because they felt they could apply skills from their discipline. Most students stated they had some prior experience or interest in global health, and sought opportunities to combine their skills and interests. One team described that a strong motivator for choosing the project was the enthusiasm shown by one of the principal investigators.

### Team Formation & Disciplinary Balance

Students were purposefully selected by faculty mentors to bridge disciplinary gaps with collaborators and achieve research goals. For example, one faculty noted a benefit of having a physician trainee on the team was that it helped the team access different resources,

“*when the team went to a particular place, for example, a hospital, just having a physician on the team opened up many doors for them, because the physician could initiate a common language.*”

Another student described an added benefit of having teams with separate disciplines was that it allowed the group to delegate tasks and work more efficiently. One student described the unique roles each team member brought to the project and how these roles contributed to the overall product:

“*I think comparing across all pros and cons coming to [country name] as a team has been more valuable for me. The single positive aspect of coming by myself is I was able to get more work done. When you’re in a team you have to move together as a cohesive group, and, so when, that was I think the only benefit of having come alone. But when I’ve come with a team, we would go through our field visit then in the evening sit down and talk to each other about it. And there would be perspectives that someone else got that I did not get and that was a learning experience. Everybody sees it through the lens of their own personal experiences, past training…so that was helpful…the fact that we have another person of [local country] origin, who spoke the language, made a big difference. It put a lot of things into cultural context. I think having someone from here who is a part of the team is very valuable.”*

Many (10/13) students mentioned that communication logistics, e.g. arranging meetings and keeping all partners and team members informed, were a challenge. One student described how her group addressed this by keeping a shared document of notes summarizing each meeting held by various team members. Another student described that each student having different schedules and program requirements limited the group’s time in country and made establishing pre- or post-travel connections and logistics difficult. Six students also described the importance of a positive relationship between the Hopkins primary faculty investigator(s) (PI) and local collaborators. One student elaborated how the PI’s history with collaborators was instrumental to daily operations, stating,

“*[the local coordinator] basically dropped [the PI’s] name and… [it was] a complete shift – and understanding who interacts with him and what’s he’s done, probably would have been helpful for us being able to leverage, because if [the local collaborator] had not been there we would not have been able to use that [relationship] … because we wouldn’t know it.*”

In contrast, students at one site described experiencing issues with communication, frequency of interactions, and defining roles between themselves, their PI, and the local collaborators made the experience challenging.

One challenge some teams faced was ensuring that different skills and knowledge were included in the process. Four students ranked being assigned tasks that allowed them to make a meaningful contribution to the project as “difficult” (reporting a score of 70 or higher). One student elaborated that she felt her discipline did not contribute to the project stated,

“*If I wasn't on the team I think the project would have been the same. I don't think that a [student’s discipline] perspective for the scope of the study was 100% necessary*.”

Some faculty explicitly tried to prevent an imbalance of disciplines when designing the team. One faculty participant addressed this by saying,

“*from each of them we had specific expectations that they would contribute to a particular piece of the bigger picture*”

Most students reported recognizing that their teammates had different skillsets and strengths intended for different project activities. At one site, a student reported this allowed them to “divide and conquer” assigned tasks. At another site, some project activities originally intended for students from specific disciplines were not available. Students there adjusted by reassigning tasks and engaging these team members in other activities that allowed them to use existing skillsets and build new ones.

### Interdisciplinary learning

Students were asked to consider the benefits and challenges of interdisciplinary teamwork. Almost every student (12/13) stated that working with students from other disciplines was valuable and forced them to consider other perspectives; as one student expressed,

*“Part of [working in team] is that you make certain assumptions and then someone will either adjust or shot down, or ask another key question that makes you question your assumption*.”

The item “Understanding concepts, theories, and methods of team members from other disciplines or areas” was consistently ranked one of the least difficult aspects of the program with an average difficulty rank of 30 out of 100 (100 is the most difficult). Students also ranked the item “Communicating concepts, theories, and methods from your discipline or area of interest to team members from other disciplines or areas” similarly. This contrasts with the item “balancing the different skills, working styles and personalities of your teammates when working on the same task”, which was the most difficult ranked item with an average score of 62 out of 100.

However, it was less clear how individual students felt the group benefited from their skills. One student who was the only member of her discipline on the team, when asked what benefits and challenges she faced with the team, stated,

“*the challenges were, in a way, feeling useful and contributing. Like I said, even when I feel like I have been most useful or like done the thing that I was supposed to do or contributed most to that side of the project, that never gets to anyone else because it notes in the back of my notebook, and there is no appropriate way to be like ‘now we're going to take time off from public health and I'm going to tell you everything I find fascinating*.”

Another student also stated he felt he did not have the same “disciplinary language” as the rest of the team and indicated frustration that the team could not understand or suggest additional work for him.

### Mentorship

Students’ view of mentors and their roles varied widely. On one team, a student leader emerged as an important mentor and driver of the project. Across all students, the majority stated that they received mentorship or advice from a faculty member or advisor in their own discipline, but that they did not interact regularly with faculty outside their discipline. One student described difficulties navigating multiple mentors; when asked whether the mentors on the project met his expectations, he described,

*“…met [expectations], maybe not…I feel like the expectations of the mentors, you know, it was too unclear to me what their involvement was and what we were expected to do…and I don’t know if they were on the same page so much…but I still think we got, we got close enough to where we needed to be by the time, thanks to, you know, one of the mentors.”*

One student shared that communication with his PI was challenging, and attributed this to not having clearly defined roles or logistics arranged before the team’s arrival in country. Other members from this team also stated that they felt frustrated by the gap between what they were told they would be doing and the more limited scope of work that was available at the site.

Overall, the mentorship received from PIs, collaborators, and/or teammates was viewed as helpful. Most stated that having a collaborator with local knowledge was invaluable. Local connections were highly valued, and one student commented on the balance that mentors need to provide in terms of personal guidance and connection facilitation, stating,

“*there has to be that right balance of you know your PI not being too over powering but at the same time being there to make sure you’re building those connections and making sure you’re on track.*”

## Discussion

The pilot year of the GEMS program successfully exposed students, faculty, and collaborators to a wide range of disciplines in global health. This program also introduced a new model of funding which included both trainee stipends and the provision of funding to partners to build site infrastructure and support staff effort. Not unexpectedly, the desire to learn how other disciplines contribute to global health solutions was a strong motivator expressed by students to join the program. Participants clearly stated the value of having multiple disciplines at the table and the opportunity to learn from each, and several students indicated that having different disciplines in the group allowed technical and personal bridges to be built which may not have been possible otherwise. Global health is inherently interdisciplinary, drawing perspectives from clinical care, population- and community-based health interventions, economics, and anthropology, among many; this program aimed to provide important training for young investigators and practitioners on how to better address these challenges in a real-world context.

Mentorship stood out as an important factor and challenge across the three teams. Two students at one site described the difficulties of finding mentorship within the team since the PI was not from their area of expertise. In his discussion of cross-discipline collaboration and communication, Jeffrey describes the importance of an “intermediary”, whose responsibility is to be “credible and competent” and to have “experience operating intellectually in more than one disciplinary area” [19]. While it may be difficult to find one person who embodies this role, engaging several faculty members to serve as informal mentors to the student team could allow for better support of student performance.

Another common challenge was interdisciplinary communication. Communication accommodation theory provides insight into the intergroup and interpersonal factors of communication which influence the success of interdisciplinary collaborations [20]. Jeffrey acknowledges the importance of personality in shaping team formation and communication and points to essential skills to support communication among different disciplines, including the recognition and formation of a shared vocabulary. Rickard points to interpersonal conflict and disagreement as one of the initial barriers to the basic team functionality [19, 21]. Our interviews reflected the importance of clear communication between PIs and team members, as many students indicated frustration with feeling comfortable in communicating the relevance of their discipline in the project.

There are several limitations to our study. Interviews occurred at very different stages during each site’s project; one team had been in country for more than a month while another arrived two weeks prior to the interview. However, respondents had completed at least 75% of their experiences at the time of interview. The responses from each team capture a different stage of cohesion and production and represent small teams, limiting our ability to generalize these findings to larger teams. Our evaluation design and analysis were not guided by an overarching framework, in part because we wanted to embrace a grounded theory methodology [22] that allows for the data to inform resulting conceptual frameworks for this program. While there is a wealth of research on team formation and cross-disciplinary communication, including the seminal model of “forming-storming-norming-performing” developed by Bruce Tuckman [23, 24], very little focuses on teams in a global, low-resource setting [25], and existing global health educational frameworks focus on the individual experience, particularly among medical and nursing trainees [26–28].

Our interviews have highlighted several challenges and enablers of interdisciplinary team formation and functioning, including identifying mentors in students’ specific disciplines, recognizing the need for students to identify and complete tasks they perceive relevant to their discipline and the group mission, and preparing in advance for logistical barriers. While some challenges, such as improving logistics planning, are changeable, many of these are manifestations of inherent differences in personalities, skills, and professional expertise that result from bringing people from different backgrounds together. We see these challenges falling into two broad categories: “Pre-team formation”, and “post-team formation”. In the “pre-team formation” phase, PIs can work to build teams of personalities and styles that might work harmoniously together, and be explicit about roles, responsibilities, and expectations. However, building a team that is wholly cohesive may not always be feasible or practical. Interdisciplinary teams should focus less on minimizing differences within the team, and instead 1) acknowledge that these differences exist and 2) identify ways they can function *despite* these differences.

Recognizing that these tensions are important sources of growth and innovation and reflecting on our experience with global health training and education, the CGH offers four recommendations to optimize team functioning for institutions and universities developing global health interdisciplinary teams.

### Recommendation 1

Address interdisciplinary communication early. Each discipline offers an important perspective and can help frame projects. It is important to introduce the team to each member’s individual skills and perspective early in the team formation process to facilitate understanding, inclusion, and mentorship. Hold interdisciplinary meetings prior to any international travel and training and engage all disciplines in the project planning process. Early intervention here can improve the pathways for bi-directional learning, so students feel like they are both contributing and receiving knowledge from the team equally.

### Recommendation 2

Develop individual and team work plans during group formation. Ground individual tasks in the overarching goals of the project. Outline each member’s responsibilities and emphasize points for disciplinary cross-over and intra-team collaboration, as well as mentorship. If needed, PIs should be willing to identify or accept supplemental mentors for students whose discipline is outside their area of expertise.

### Recommendation 3

Provide a space for team members to meet as individuals prior to departure. Faculty need to consider the unique personalities and perspectives of each team member, and encourage space for members to learn about each other independent of their skillsets or roles in the team. These opportunities for personal connection could help foster the development of a “psychological safety net” that is necessary for creativity and innovation [14].

### Recommendation 4

Regular check in and communication with faculty advisors and local investigators/collaborators is vital. Strong relationships between faculty and collaborators are a catalyst to project progression and success. Students rely on this relationship and their own relationships with mentors for personal and professional growth.

## Conclusions

Our work provides a reflection on interdisciplinary team experiences in a changing global context, and lays the pathway for new avenues of research. Programs offered by different universities and institutions will each have their own objectives and challenges. The recommendations to 1) address interdisciplinary communication, 1) develop workplans during team formation, 3) provide a space for members to meet prior to departure, and to 4) conduct regular check-ins are intended to be launching points for consideration when addressing a program’s specific concerns. Global health education in recent years has focused on the identification of core competencies and structures that promote the development of these competencies [29], but largely in the context of single-disciplinary experiences. Further evaluation of the GEMS sites could identify innovative strategies to foster these competencies which may not be possible through the individual experience.
